# Ba_4_Al_7_Li_28.08_O_26.92_N_1.08_, the Barium Oxonitridolithoaluminate with a Highly Condensed LiO_4_ Tetrahedra Framework

**DOI:** 10.1021/acs.inorgchem.2c03211

**Published:** 2022-12-16

**Authors:** Daniel S. Wimmer, Markus Seibald, Dominik Baumann, Klaus Wurst, Hubert Huppertz

**Affiliations:** † Institut für Allgemeine, Anorganische und Theoretische Chemie, Universität Innsbruck, Innrain 80-82, A-6020 Innsbruck, Austria; ‡ ams-OSRAM International GmbH, Mittelstetter Weg 2, D-86830 Schwabmünchen, Germany

## Abstract

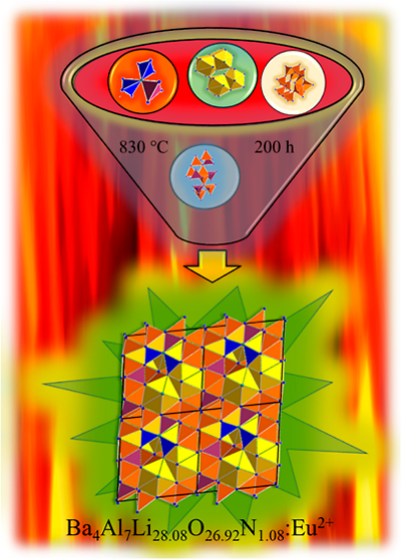

The new compound Ba_4_Al_7_Li_28.08_O_26.92_N_1.08_ consists of AlO_4_/AlO_3_N tetrahedra, 10-fold coordinated Ba^2+^ cations, and a highly condensed edge- and corner-sharing LiO_4_ tetrahedra framework, which leads to a degree of condensation greater than 1. The first barium oxonitridolithoaluminate was synthesized by a high-temperature solid-state reaction in a weld-shut tantalum ampoule and the crystal structure has been determined by single-crystal X-ray diffraction. Ba_4_Al_7_Li_28.08_O_26.92_N_1.08_ crystallizes in the monoclinic space group *P*2_1_/*m* (no. 11) with the lattice parameters *a* = 1052.41(3), *b* = 615.93(2), *c* = 1088.45(4) pm, β = 98.86(1)°, and a volume of *V* = 0.69712(4) nm^3^. In addition, Ba_4_Al_7_Li_28.08_O_26.92_N_1.08_ doped with the activator ion Eu^2+^, exhibits a broad band emission with a maximum at λ_max_ = 524 nm (2.34 eV) with a fwhm of 112 nm (4373 cm^–1^/0.54 eV), which can be described by a superposition of two adjusted emission bands at λ_max_ = 515 nm (2.41 eV) with a fwhm of 70 nm (2704 cm^–1^/0.34 eV), and at λ_max_ = 574 nm (2.18 eV) with a fwhm of 127 nm (4127 cm^–1^/0.51 eV).

## Abstract 2

The compound Ba_4_Al_7_Li_28.08_O_26.92_N_1.08_:Eu^2+^ was synthesized by a high-temperature solid-state reaction. The anionic network in the structure consists of AlO_3_N/AlO_4_ and highly condensed edge- and corner-sharing LiO_4_ tetrahedra resulting in a degree of condensation of ∼1.25. The 10-fold coordinated Ba^2+^ cations are incorporated between seven-membered rings of the anionic framework and form endless chains along the crystallographic *b*-axis. The new compound could be successfully doped with the activator ion Eu^2+^ exhibiting interesting luminescence properties.

## Introduction

Because the field of oxonitridolithoaluminates is still a fairly new substance class, there are not yet many known compounds in which exclusively Li(O,N)_4_ and Al(O,N)_4_ tetrahedra form the anionic framework structure. The best-known representatives include compounds such as Sr[Li_2_Al_2_O_2_N_2_]:Eu^2+^^1^ and Sr[Li_2.5_Al_1.5_O_3_N]:Eu^2+^,^2^ where mainly Sr^2+^ cations are incorporated in the *vierer* ring^3^ channels. Up to now, no compound is known in which Ba^2+^ cations fill the channels formed by the anionic framework structure. Extending the field to include the substance classes of pure oxide and nitride variants, the first and only barium lithoaluminate with the sum formula Ba_3_Li_7_Al_3_O_11_ was published by Nishita and Yamane in 2021.^4^ In the last years, variants with other alkaline earth cations such as Ca[LiAl_3_N_4_]:Eu^2+^,^5^ Ca[LiAlN_2_],^6^ Ca_18.75_[Li_10.5_Al_39_N_55_],^7^ Sr_4_[LiAl_11_N_14_]:Eu^2+^,^8^ Sr[LiAl_3_N_4_]:Eu^2+^,^9^ Sr_2_LiAlO_4_:Eu^2+^,^10,11^ and Sr_0.833_Al_1.167_Li_2.833_O_4_:Eu^2+^^12^ could be synthesized and characterized. Additionally, most of these phases could successfully be doped with the activator ion Eu^2+^ showing interesting luminescence properties in the yellow to red spectral region. These compounds form a highly condensed anionic framework, which is discussed to be favorable for phosphors in relation to a small Stokes shift, less electron-phonon coupling, and a favorable thermal-quenching behavior.^13,14^

The degree of condensation κ is defined by the ratio of tetrahedral centers *T* and coordinating anions N/O [κ = *n*(*T*)/*n*(N,O)]. In relation to binary nitrides and oxides of elements such as Si, Al, Be, and Li, the degree of condensation increases with the formal substitution of O by N and with the decrease in oxidation number of the tetrahedral center κ(Si_3_N_4_) = 0.75, κ(SiO_2_) = 0.5, κ(AlN) = 1, κ(β-Be_3_N_2_) = 1.34, κ(BeO) = 1, and κ(Li_2_O) = 2. Although for most silicate and aluminate compounds, there is no known degree of condensation greater than 1 due to the fact that the Al(O,N)_4_ and Si(O,N)_4_ tetrahedra are exclusively edge- and corner-shared among each other. In the case of beryllates, there are some representatives such as Sr[Be_6_ON_4_]:Eu^2+^ (κ = 1.2),^15^*M*Be_20_N_14_:Eu^2+^ (*M* = Sr, Ba) (κ = 1.42),^16^ and Eu_3_Be_22_N_16_O (κ = 1.29)^17^ exhibiting a degree of condensation greater than 1. The structure of the mentioned beryllates is characterized by Be(O,N)_4_ tetrahedra, which are connected over several edges and corners, and in the compound Eu_3_Be_22_N_16_O additionally by trigonal planar BeN_3_ units. The lower oxidation state of beryllium enforces an increase of tetrahedral centers, which leads necessarily to a higher degree of condensation. In the field of (oxonitrido)lithoaluminates, KLi_4_AlO_4_ (κ = 1.25)^18^ is the only known representative with a degree of condensation greater than 1. The anionic framework in KLi_4_AlO_4_ is built up by “isolated” AlO_4_ tetrahedra, which frequently share common corners and edges with LiO_4_ tetrahedra.

Here, we present the crystal structure and luminescence properties of a new oxonitridolithoaluminate possessing the composition Ba_4_Al_7_Li_28.08_O_26.92_N_1.08_ and with a highly condensed LiO_4_ tetrahedra framework resulting in a degree of condensation of ∼1.25.

## Results and Discussion

### Crystal Structure

The compound Ba_4_Al_7_Li_28.08_O_26.92_N_1.08_ crystallizes in the monoclinic space group *P*2_1_/*m* (no. 11) with the lattice parameters *a* = 1052.41(3), *b* = 615.93(2), *c* = 1088.45(4) pm, β = 98.86(1)°, and a volume of *V* = 0.69712(4) nm^3^. Details on the crystal structure refinement and atomic coordinates are given in Tables 1 and 2. Displacement parameters and interatomic distances are listed in Tables S1 and S2 in the Supporting Information.

**Table 1:** Crystal Data and Structure Refinement of Monoclinic Ba_4_Al_7_Li_28.08_O_26.92_N_1.08_

empirical formula	Ba_4_Al_7_Li_28.08_O_26.92_N_1.08_
molar mass/g mol–1	1378.95
crystal system	monoclinic
space group	*P*2_1_/*m*
single-crystal data	
single-crystal diffractometer	Bruker D8 Quest Kappa
radiation	Mo-Kα (λ = 71.073 pm)
*a*/pm	1052.41(3)
*b*/pm	615.93(2)
*c*/pm	1088.45(4)
β/deg	98.86(1)
*V*/nm^3^	0.69712(4)
formula units per cell Z	1
calculated density/g cm^–3^	3.285
crystal size/mm^3^	0.080 × 0.050 × 0.030
temperature/K	299(2)
detector distance/mm	50
exposure time	0.5°/frame; 40 s/frame
absorption coefficient/mm^–1^	5.905
*F*(000)/e	622
θ-range/deg	5.012–75.454
range in *hkl*	±15, ±9, ±16
reflections total/independent	16044/2723
reflections with*I* ≥ 2σ(*I*)	2595
*R*_σ_	0.0195
data/ref. parameters	2723/186
absorption correction	multi-scan
final *R*_1_/*wR*_2_ [*I* ≥ 2σ(*I*)]	0.0180/0.0432
final *R*_1_/*wR*_2_ (all data)	0.0192/0.0436
goodness of fit on *F*^2^	1.106
largest diff. peak/hole/e·Å^–3^	1.348/–1.55

**Table 2:** Wyckoff Positions, Atomic Coordinates, and Equivalent Isotropic Displacement Parameters *U*_eq_ (Å^2^) of Ba_4_Al_7_Li_28.08_O_26.92_N_1.08_ (Standard Deviations in Parentheses)

atom	Wyckoff-position	*x*	*y*	*z*	*U*_eq_	occ.
Ba1	2*e*	0.50604(2)	1/4	0.22232(2)	0.01221(4)	1
Ba2	2*e*	0.82241(6)	3/4	0.63786(6)	0.0105(2)	0.48
Ba3	2*e*	0.8112(2)	3/4	0.6495(2)	0.0095(3)	0.2
Ba4	4*f*	0.81349	0.7930(2)	0.63677(8)	0.0080(2)	0.16
Al1	2*e*	0.62315(6)	3/4	0.38083(5)	0.0058(2)	1
Al2	2*e*	0.31898(6)	3/4	0.23185(5)	0.0055(2)	1
Al3	2*e*	0.92364(6)	1/4	0.15157(6)	0.0054(2)	0.94
Al4	2*e*	0.44923(9)	1/4	0.90836(9)	0.0057(2)	0.53
Al5	2*e*	0.2141(3)	3/4	0.5342(3)	0.0111(5)	0.03
Li1	4*f*	0.6810(2)	0.0349(5)	0.0089(2)	0.0107(5)	1
Li2	4*f*	0.7691(3)	0.5176(4)	0.2239(2)	0.0111(5)	1
Li3	2*e*	0.92364(6)	1/4	0.15157(6)	0.0054(2)	0.06
Li4	2*e*	0.44923(9)	1/4	0.90836(9)	0.0057(2)	0.47
Li5	2*e*	0.2141(3)	3/4	0.5342(3)	0.0111(5)	0.97
Li6	2*e*	0.0728(4)	3/4	0.3310(4)	0.0134(7)	1
Li7	4*f*	0.0826(3)	0.5332(5)	0.0951(3)	0.0125(5)	1
Li8	4*f*	0.9247(3)	0.9887(4)	0.4080(2)	0.0097(4)	1
Li9	4*f*	0.3970(4)	0.9285(7)	0.4765(4)	0.0219(8)	0.82
Li10	2*e*	0.0911(4)	1/4	0.8646(4)	0.0111(7)	1
Li11	2*e*	0.2178(5)	1/4	0.0671(4)	0.0123(8)	0.9
O1	2*e*	0.4941(3)	3/4	0.2517(2)	0.0143(3)	0.46
O2	2*e*	0.5582(2)	3/4	0.5263(2)	0.0119(3)	1
O3	2*b*	1/2	0	0	0.0128(3)	1
O4	2*e*	0.7543(2)	1/4	0.1279(2)	0.0087(2)	1
O5	2*e*	0.2716(2)	1/4	0.8988(2)	0.0092(3)	1
O6	2*e*	0.0202(2)	3/4	0.4956(2)	0.0088(2)	1
O7	4*f*	0.9741(2)	0.4953(3)	0.23361(9)	0.0082(2)	1
O8	4*f*	0.2618(2)	0.5083(2)	0.1530(2)	0.0097(2)	1
O9	2*e*	0.0092(2)	1/4	0.0200(2)	0.0087(2)	1
O10	2*e*	0.2638(2)	3/4	0.3759(2)	0.0150(3)	1
O11	4*f*	0.7200(2)	0.5145(3)	0.3861(2)	0.0143(2)	1
N1	2*e*	0.4941(3)	3/4	0.2517(2)	0.0143(3)	0.54

**Figure 1:**
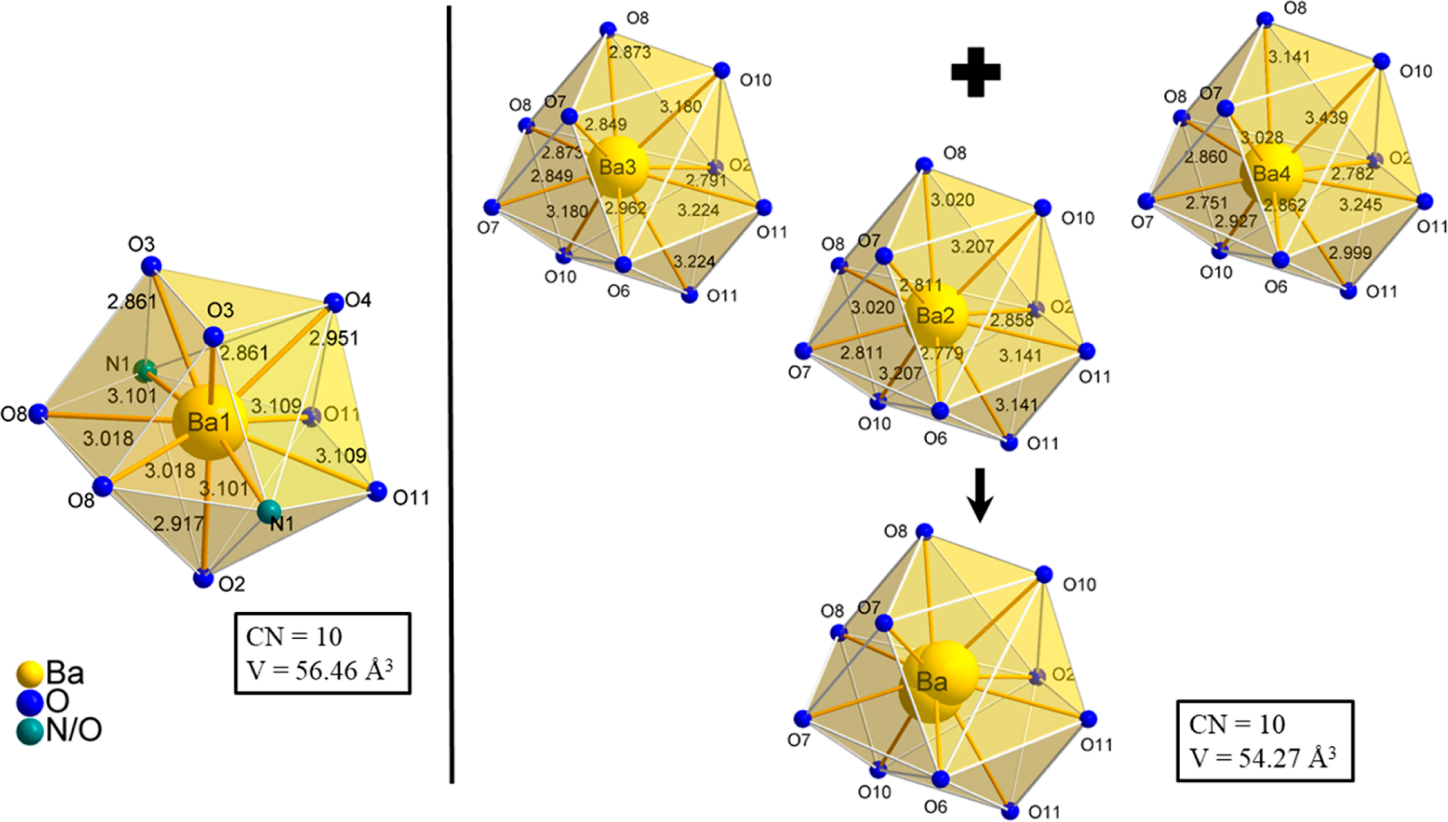
In the left half, the coordination polyhedron including the Ba1–O/N distances of the Ba1 site and the resulting polyhedron size is represented. In the right half, the coordination polyhedron including the Ba–O/N distances of the split positions Ba2, Ba3, and Ba4 and the resulting polyhedron size is shown.

The crystal structure of the compound Ba_4_Al_7_Li_28.08_O_26.92_N_1.08_ is built up by two crystallographically distinguishable barium sites, which are coordinated tenfold by O^2–^ and N^3–^ anions, respectively. Based on the high-quality single-crystal data set, there are indications to distinguish between N and O on the anion positions. The O2 to O11 sites are fully occupied by O and show minimal deviation from the occupancy of 1 by setting them free in the refinement. By refining N on these sites, this leads to a significant overoccupation. In comparison, the mixed occupied N1/O1 site exhibits a significant under- or overoccupation, respectively, in the case of a pure occupation of O and N. The resulting anionic displacement parameters of this site show a relatively large deviation in relation to the other fully occupied O2–O11 sites, indicating a mixed occupied site of N and O. In addition, a better *R*-value was also observed when this site is mixed occupied. Furthermore, the connectivity of the different sites as well as the resulting bond lengths compared with compounds known from literature confirms the assignment. Because the N1/O1 site is mixed occupied by 54% N and 46% O, three different coordination spheres result for the Ba1 site. The first possibility is that the Ba1 site is coordinated by 10 O^2–^ anions. In the second case, the Ba1 site would be coordinated by nine O^2–^ and one N^3–^ anions, and the third possibility would be that the Ba1 site forms a coordination sphere to eight O^2–^ and two N^3–^ anions. The Ba1–O distances range from 286.07(2) to 310.9(2) pm and the Ba1–O1/N1 distance is 310.07(4) pm. The Ba2 site is exclusively coordinated by 10 O^2–^ anions and their Ba2–O distances vary between 277.9(2) and 320.71(5) pm. Due to the high displacement of the Ba^2+^ cations of the Ba2 site and the relatively high residual electron density around this position, split positions were introduced in the single-crystal refinement and are additionally described by the Ba3 and Ba4 site. Due to the spatial proximity and to avoid a strong correlation between the Ba2, Ba3, and Ba4 sites, only an isotropic refinement of the displacement parameters was applied. Considering the Ba3 and Ba4 site, the Ba3–O distances are between 279.1(2) and 322.4(2) pm, while the Ba4–O distances range from 275.1(2) to 324.5(2) pm. The Ba–O/N distances of the Ba1 and Ba2 site, including the split position Ba3 and Ba4 site, correspond well with the sum of the ionic radii and are in good accordance with Ba–O/N distances known from literature.^19−23^ The described split positions above are then referred to as the Ba2 site in the following. The polyhedra of the Ba1 and Ba2 sites could be described as distorted single-capped triangular cupola, which polyhedron shape could also be observed in compounds such as Eu_3_Be_22_N_16_O.^17^ The program VESTA 3(24) was used to calculate the polyhedron size (see Figure 1).

**Figure 2:**
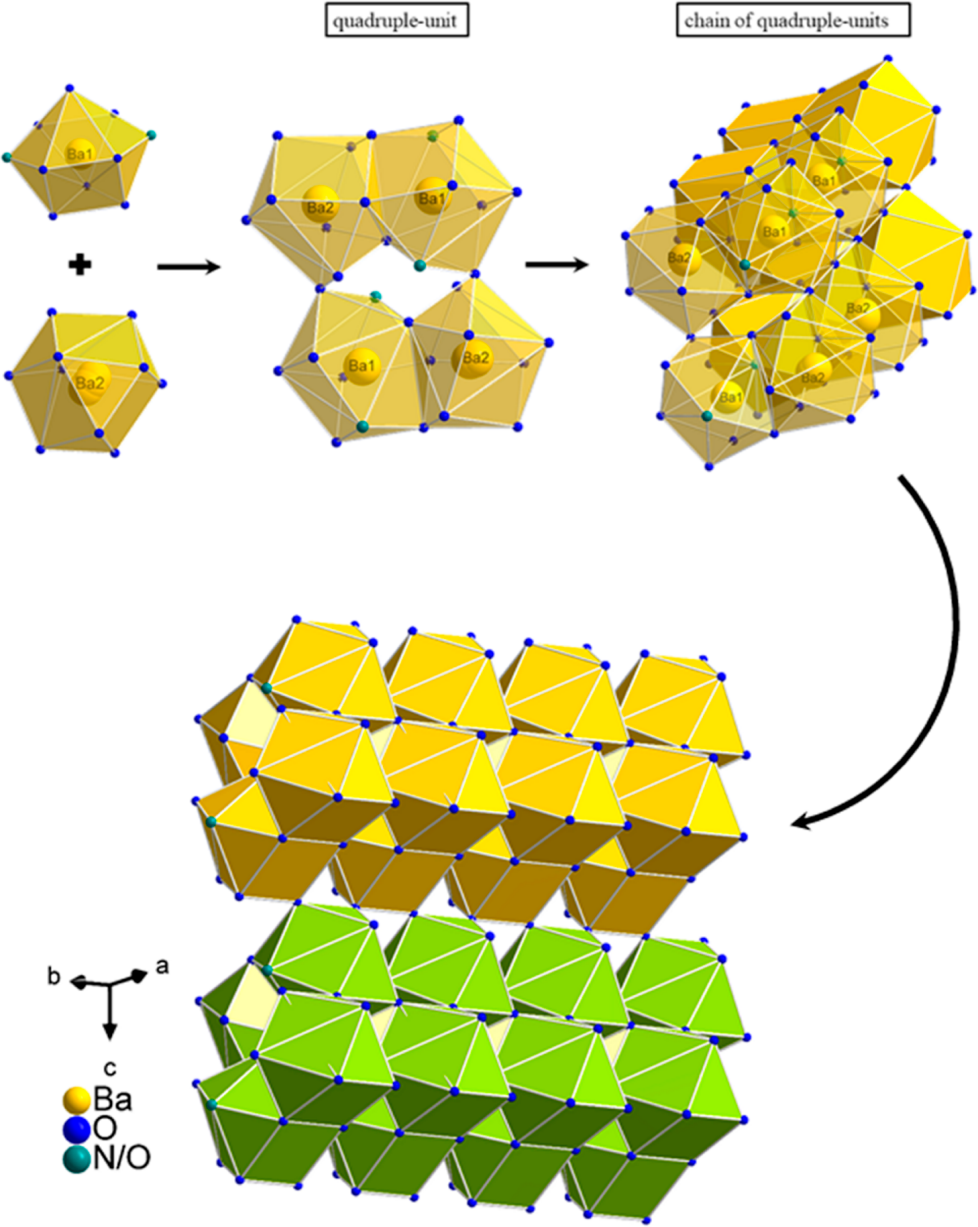
In the upper half, the quadruple-units, formed from the Ba1 and Ba2 polyhedra (yellow), as well as the formed chain from the quadruple-units along the crystallographic *b*-axis is represented. In the lower half, the connection of the quadruple-unit chains along the crystallographic *c*-axis is shown (one chain is displayed in yellow and one is displayed in lime).

In the structure of Ba_4_Al_7_Li_28.08_O_26.92_N_1.08_, quadruple-units, consisting of two Ba1 and two Ba2 polyhedra, are formed and they are built up in such a way that one Ba1 polyhedron is face-shared to one Ba2 polyhedron via the triangular face of the single-capped triangular cupola as well as corner-shared to another Ba2 polyhedron via the O^2–^ anion, and vice versa. These quadruple-units then build chains along the crystallographic *b*-axis, so that the Ba1 polyhedra are each corner-shared to one another of the previous and next quadruple-unit via the mixed occupied N1/O1 site and share a common corner with the Ba2 polyhedra of the next quadruple-unit via the O^2–^ anion. In relation to the Ba2 polyhedra, they are each corner-shared to one another of the previous and next quadruple-unit via an O^2–^ anion and share a common corner with the Ba1 polyhedra of the next quadruple-unit via the O^2–^ anion (see Figure 2). In addition, the chains of quadruple-units are corner-shared via the O^2–^ anion of the Ba1 polyhedra along the crystallographic *c*-axis.

**Figure 3:**
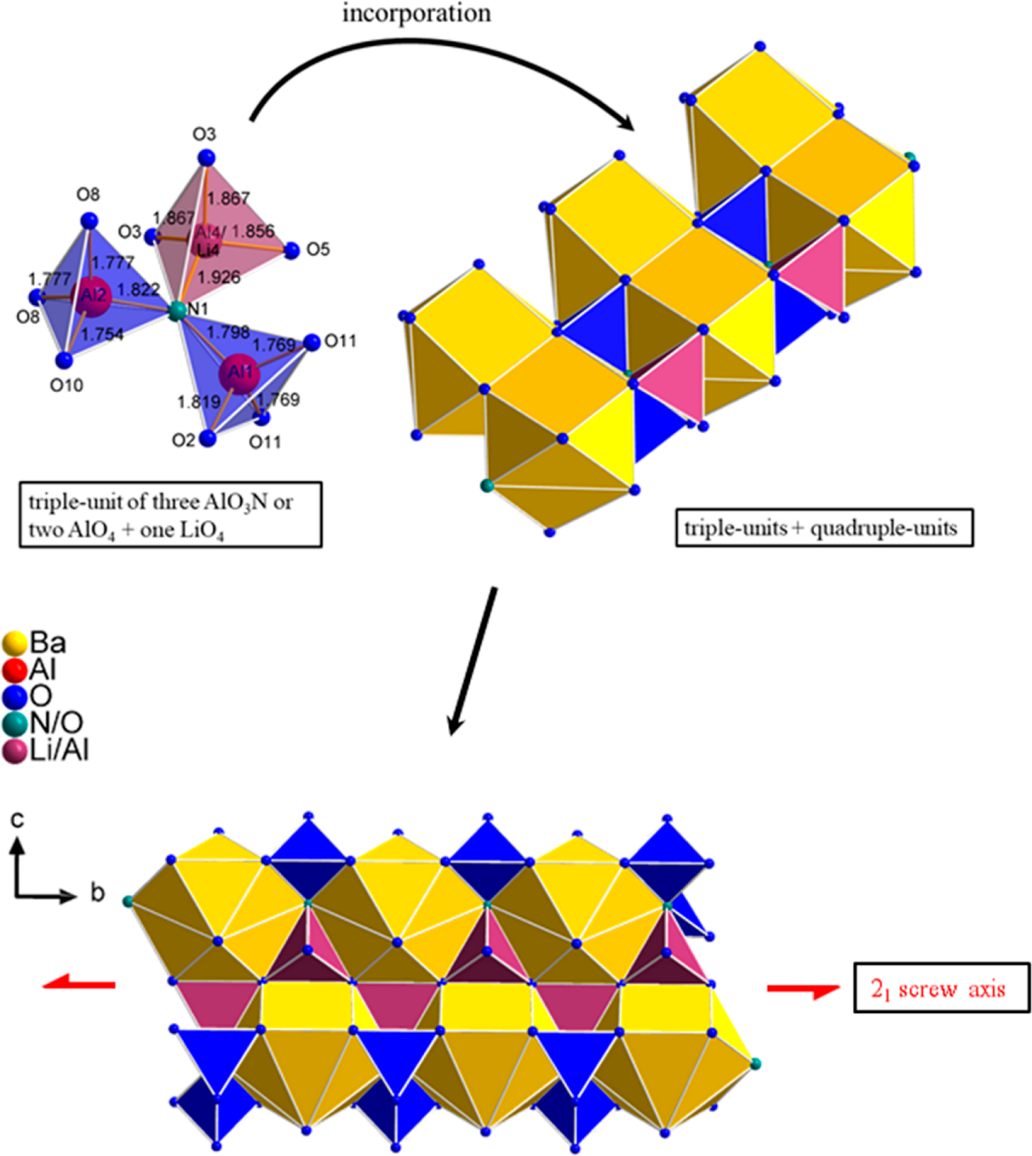
On the upper left, the connection within the triple-units via the mixed occupied O1/N1 site is represented. On the upper right, the incorporation of the triple-units in the quadruple-unit chains, built up by Ba1 and Ba2 polyhedra, is shown. In the lower half, the 2_1_ screw axis is clearly visible, which results from the special arrangement of the triple-units, including the Ba1 polyhedra (the Ba2 polyhedra have been omitted in the lower graphic for a better overview). The Ba1 and Ba2 polyhedra are displayed in yellow, the AlO_3_N/AlO_4_ tetrahedra are displayed in blue, and the mixed occupied LiO_4_/AlO_4_/AlO_3_N tetrahedra are displayed in plum.

Furthermore, units consisting of three AlO_3_N tetrahedra or two AlO_4_ plus one LiO_4_ tetrahedra, respectively, are formed by two pure Al sites (Al1 and Al2) as well as from the Li4/Al4 site, where the latter is occupied by about 53% with Al and 47% with Li. Mixed occupied sites of Al and Li have also been observed in other compounds known from literature such as Sr_4_[LiAl_11_N_14_]:Eu^2+^.^8^ Depending on the occupation of the Li4/Al4 site and the resulting occupation of the N1/O1 site, this unit is arranged in such a way that a planar triple-unit of AlO_3_N tetrahedra or of two AlO_4_ plus one LiO_4_ tetrahedra is formed, which are corner-shared via the N^3–^ and via the O^2–^ anion, respectively. The Al1–O and Al2–O distances range from 176.9(2) to 181.9(3) pm and from 175.4(2) to 177.7(2) pm, while the Al1–O1/N1 distance is 179.8(2) pm and the Al2–O1/N1 distance is 182.2(3) pm, which are in good accordance with the sum of ionic radii and Al–O/N distances known from literature.^1,9,10^ With respect to the mixed occupation of the Li4/Al4 site, the Li4/Al4–O distances range from 185.6(2) to 186.74(5) pm and the Li4/Al4–O1/N1 distance is 192.6(2) pm corresponding to the average Al–O/N and Li–O/N distances of a pure Al and Li site (average Al2–O distances of 176.9 pm and average Li1–O distances of 195.8 pm). These triple-units are then sandwiched between the Ba1 polyhedra of the Ba polyhedra chains in such a way that each AlO_3_N/AlO_4_ or LiO_4_ tetrahedron is connected to the Ba1 polyhedra by a common edge, and in addition, the AlO_3_N/AlO_4_ tetrahedra of the Al1 site are face-shared to the Ba2 polyhedra and those of the Al2 site are edge-shared to the Ba2 polyhedra. The planar triple-units among themselves are corner-shared via the LiO_4_/AlO_3_N tetrahedron, each via two O^2–^ anions, and arrange along the crystallographic *b*-axis in the form of a 2_1_ screw axis (see Figure 3).

**Figure 4:**
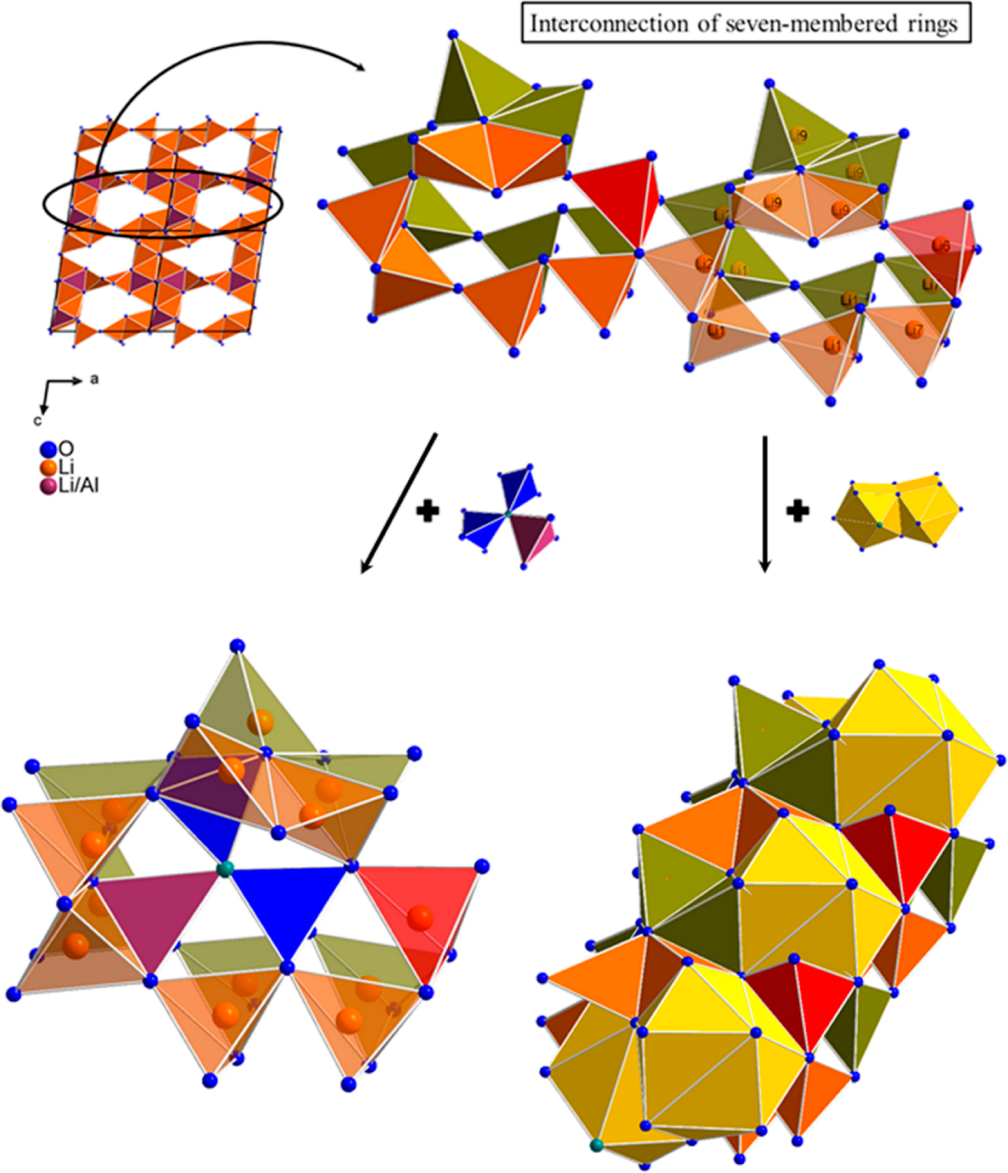
On the upper left, the channel within the seven-membered ring in a 2 × 2 × 2 super cell is shown. In the upper right, the structure of a seven-membered ring unit consisting of two identical seven-membered rings and their interconnection is represented. The first seven-membered ring is formed by the orange LiO_4_ tetrahedra and the second by the olive LiO_4_ tetrahedra. The red LiO_4_ tetrahedra reflect the Li6 site and are part of both the orange and the olive seven-membered rings. On the lower half, the incorporation of the planar triple-unit in a unit of two seven-membered rings as well as the connection of the seven-membered rings and the Ba1 and Ba2 polyhedra is illustrated. The Ba1 and Ba2 polyhedra are displayed in yellow, the AlO_3_N/AlO_4_ tetrahedra are displayed in blue, and the mixed occupied LiO_4_/AlO_4_/AlO_3_N tetrahedra are displayed in plum.

As derived from the sum formula Ba_4_Al_7_Li_28.08_O_26.92_N_1.08_, the degree of condensation κ = *n*(Li, Al)/*n*(O, N) is ∼1.25, resulting in a highly condensed anionic framework. This is due to the fact that the proportion of Li^+^ cations in the structure is much higher than that of the Al^3+^ cations and, compared to (oxo)nitridosilicates or -aluminates, the Li^+^ cations in the form of LiO_4_ tetrahedra can be connected over several edges due to the lower formal charge of Li^+^ compared to Al^3+^ and Si^4+^. Due to the high electrostatic repulsion of the Al^3+^ and Si^4+^ cations resulting from the high formal charge, the tetrahedra are connected to each other only via common corners and edges because the tetrahedral centers would come too close to each other in case of face-sharing. A degree of condensation greater than 1 can already be observed in (oxo)nitridoberyllates such as Eu_3_Be_22_N_16_O^17^ because the formal charge of Be^2+^ is also smaller than that of Al^3+^ and Si^4+^ here. The highly condensed LiO_4_ framework in the structure of the title compound is built up by eight crystallographically distinguishable Li sites and two sites mixed occupied by Al and Li. Along the crystallographic *b*-axis, a channel within the seven-membered ring is formed, where the seven-membered ring is exclusively built up by corner- and edge-sharing LiO_4_ tetrahedra of the Li1, Li2, Li6, Li7, and Li9 sites. On the one hand, the seven-membered rings are identically arranged and are corner-shared to each other via the O^2–^ anions except for the LiO_4_ tetrahedra of the Li9 site, which are edge-shared via two O^2–^ anions, but on the other hand, two seven-membered rings share the LiO_4_ tetrahedron of the Li6 site. The single-crystal refinement shows that the Li9 site is only occupied by about 80% (occ. = 0.82) and although every fifth LiO_4_ tetrahedron of the Li9 site is absent in the structure, the structure of the seven-membered rings is maintained due to the high interconnection of the LiO_4_ tetrahedra of the Li9 site. A unit of two seven-membered rings, sharing the LiO_4_ tetrahedron of the Li6 site, arrange themselves in the structure so that they enclose the planar triple-unit consisting of three AlO_3_N tetrahedra or two AlO_4_ plus one LiO_4_ tetrahedra, respectively, by sharing common corners and edges. In addition, the LiO_4_ tetrahedra of the seven-membered ring are also edge-shared to the polyhedra of the Ba1 and Ba2 sites (see Figure 4).

**Figure 5:**
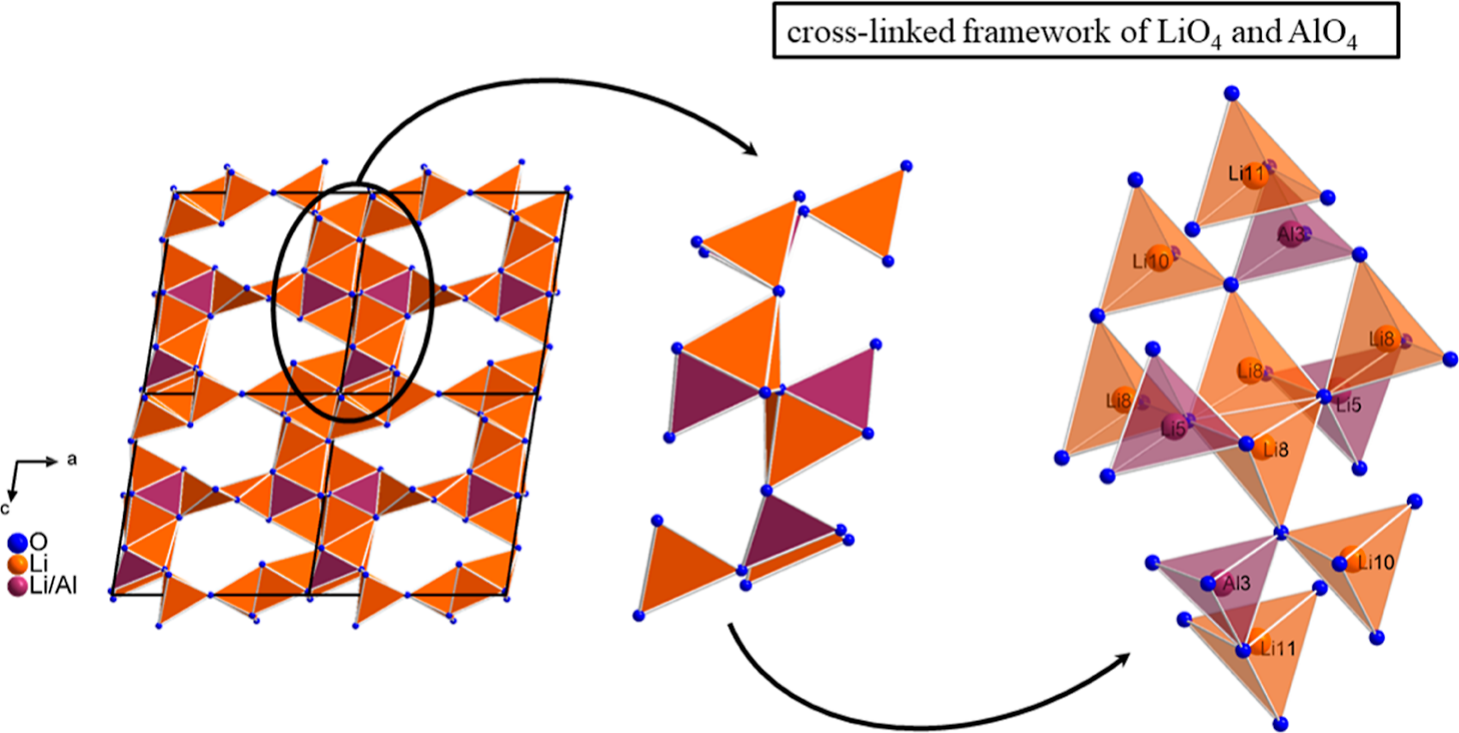
Cross-linked framework, formed by the LiO_4_ and AlO_4_ tetrahedra of three Li sites (Li8, Li10, and Li11) and mixed occupied sites (Li3/Al3 and Li5/Al5), in the structure of Ba_4_Al_7_Li_28.08_O_26.92_N_1.08_ is shown. The LiO_4_ tetrahedra are displayed in orange and the mixed occupied LiO_4_/AlO_4_ tetrahedra are displayed in plum.

Along the crystallographic *b*-axis, the seven-membered rings are, on the one hand, corner-shared via the LiO_4_ tetrahedra of the Li2, Li6, and Li7 site through a common O^2–^ anion (see Figure 4) and, on the other hand, these are still connected by a strongly cross-linked framework of LiO_4_ and AlO_4_ tetrahedra via corner- and edge-sharing. The cross-linked framework is built up by the LiO_4_ and AlO_4_ tetrahedra of three pure Li sites (Li8, Li10, and Li11) and by two sites, which are mixed occupied by Al and Li. According to the single-crystal refinement, the Li3/Al3 site is Al-rich and is occupied by 94% Al and only 6% Li, while the Li5/Al5 forms a Li-rich site and is occupied by 97% Li and only 3% Al. Indications for a mixed occupation are the large anisotropic displacement parameters for pure Li or Al sites and the average Li3/Al3–O distances of 178.8 pm and Li5/Al5–O distances of 193.6 pm, which are between those of a pure Al (average Al2–O distances of 176.9 pm) and Li site (average Li1–O distances of 195.8 pm), for example. Additionally, the Li11 site is only 90% occupied by Li and hence every 10th LiO_4_ tetrahedron of this site is consequently not present in the structure. The cross-linked framework is formed by corner- and edge-shared LiO_4_ and AlO_4_ tetrahedra (see Figure 5). The Li–O distances of all eight crystallographically distinguishable Li sites range from 186.7(3) to 217.7(5) pm and are in good accordance with the sum of the ionic radii and Li–O distances known from literature.^10,25−27^

**Figure 6:**
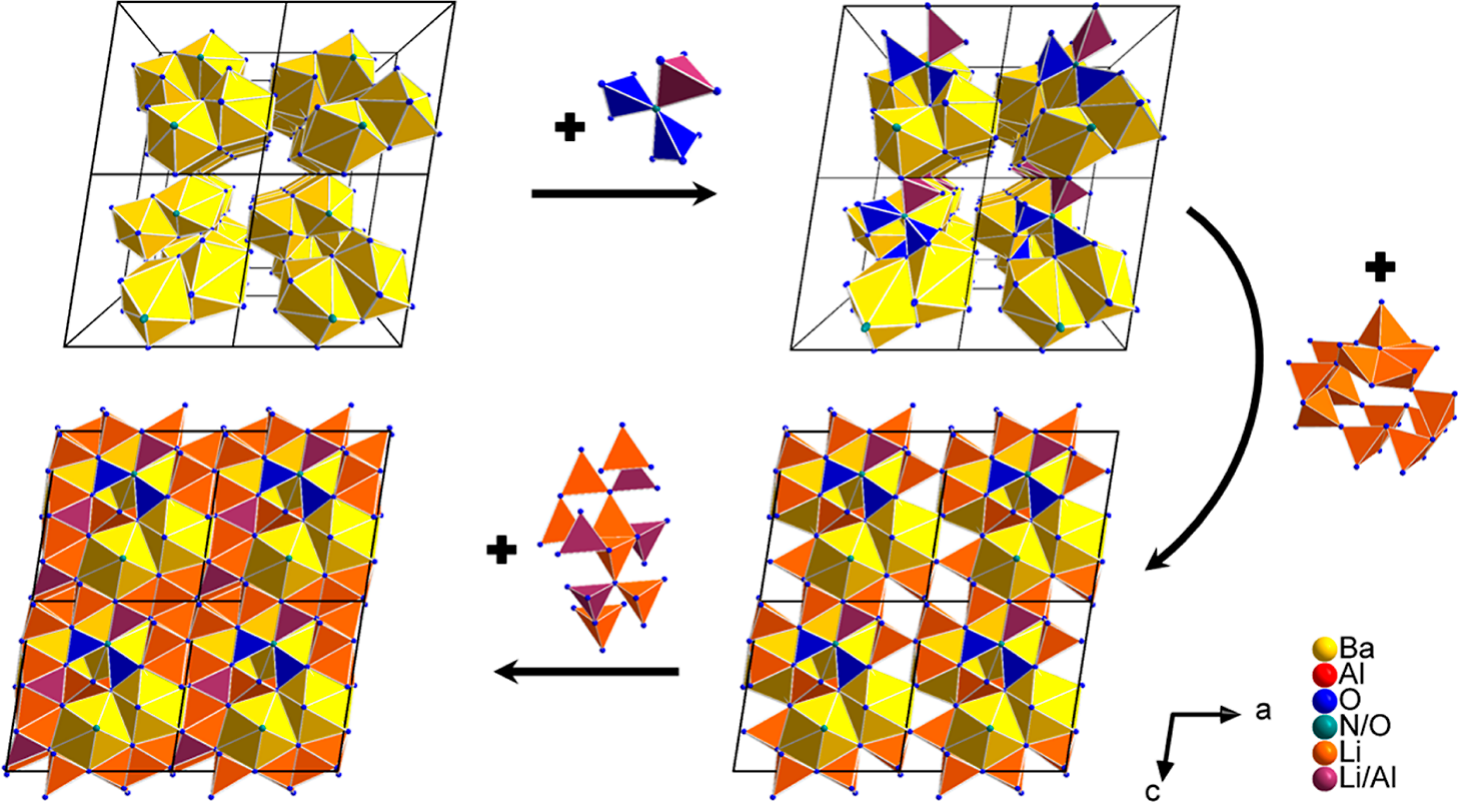
Constitution of the entire structure of Ba_4_Al_7_Li_28.08_O_26.92_N_1.08_. The Ba1 and Ba2 polyhedra are displayed in yellow, the AlO_3_N/AlO_4_ tetrahedra are displayed in blue, the mixed occupied LiO_4_/AlO_4_/AlO_3_N tetrahedra are displayed in plum, and the LiO_4_ tetrahedra are displayed in orange.

The entire structure of Ba_4_Al_7_Li_28.08_O_26.92_N_1.08_ can be summarized into four building blocks, namely, the quadruple-units of 10-fold coordinated Ba polyhedra forming a chain along the crystallographic *b*-axis, the planar triple-units consisting of AlO_3_N tetrahedra or of two AlO_4_ plus one LiO_4_ tetrahedra, respectively, the units of two seven-membered rings formed from LiO_4_ tetrahedra, and the cross-linked framework built up from AlO_4_ and LiO_4_ tetrahedra. An overview of the structure of Ba_4_Al_7_Li_28.08_O_26.92_N_1.08_ is shown in Figure 6.

The compound Ba_4_Al_7_Li_28.08_O_26.92_N_1.08_ could be successfully doped with the activator ion Eu^2+^ and the luminescence properties are discussed in the next section.

### Luminescence

**Figure 7:**
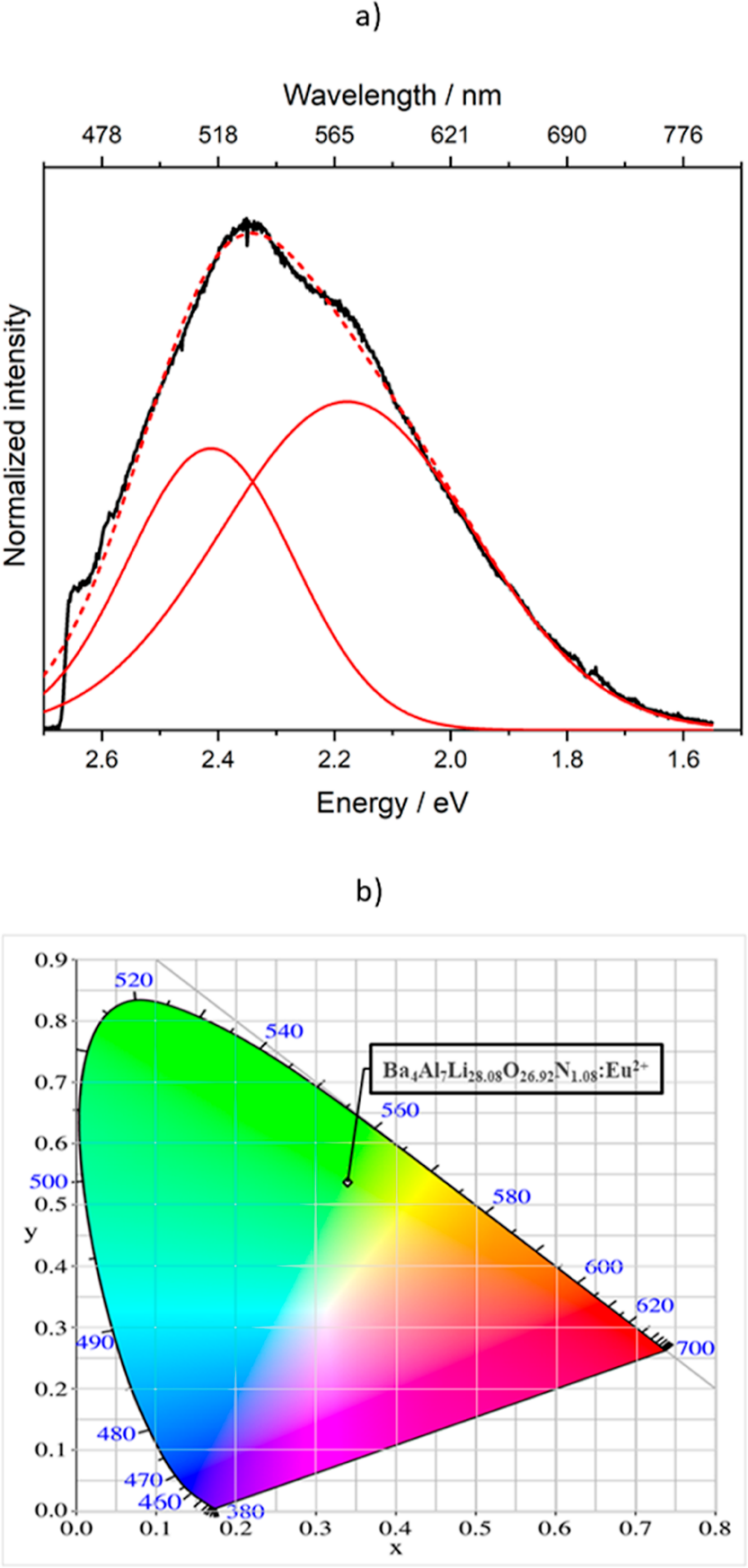
(a) Single-crystal emission spectrum of Ba_4_Al_7_Li_28.08_O_26.92_N_1.08_:Eu^2+^ (black line, experimental cut off due to laser-based setup) in combination with two adjusted Gaussian curves (red lines) and the sum of the two adjusted Gaussian curves (red dashed line). (b) Color point of Ba_4_Al_7_Li_28.08_O_26.92_N_1.08_:Eu^2+^ in the CIE-diagram.^28^

The luminescence properties of the title compound (doped with a nominal activator concentration of 2 mol % Eu^2+^) were examined on a single-crystal. Ba_4_Al_7_Li_28.08_O_26.92_N_1.08_:Eu^2+^ can be excited by near-UV to blue light and exhibits an emission in the green to yellow spectral region with a maximum at λ_max_ = 524 nm (2.34 eV) and a fwhm of 112 nm (4373 cm^–1^/0.54 eV). The measured emission profile can be described by a superposition of two adjusted emission bands at λ_max_ = 515 nm (2.41 eV) with a fwhm of 70 nm (2704 cm^–1^/0.34 eV), and at λ_max_ = 574 nm (2.18 eV) with a fwhm of 127 nm (4127 cm^–1^/0.51 eV). In the CIE-*xy* color space, this corresponds to the value *x* = 0.337(1) and *y* = 0.523(1) (see Figure 7).

The observed double band emission in Figure 7a, which can be described by a superposition of two Gaussian curves, indicates that there are two possible positions, that is, sites with significant different coordination spheres, which can be occupied by the activator ion Eu^2+^. In the structure of Ba_4_Al_7_Li_28.08_O_26.92_N_1.08_, the Ba1 site on the one hand and the sum of split positions (Ba2, Ba3, and Ba4 site) in the other hand could be suitable to host the activator ion Eu^2+^. In the case of the Ba1 site, the Ba^2+^ cation is coordinated by 10 anions. Which coordination sphere is formed around this Ba^2+^ cation depends on the occupation of the O1/N1 site, which is occupied by 53% N and 47% O. Consequently, this coordination sphere can vary between 10 O^2–^ anions and 8 O^2–^ plus 2 N^3–^ anions, which has an effect on the position of the emission band and the fwhm. The Ba1–O distances range from 286.07(2) to 310.9(2) pm, whereby the Ba1–O1/N1 distance is 310.07(4) pm, resulting in a polyhedron size of 56.46 Å^3^. The split position of the Ba2 site exclusively forms a coordination sphere to 10 O^2–^ anions resulting in a polyhedron size of 54.27 Å^3^. Considering the Ba3 and Ba4 site, additionally describing the split position, the Ba–O distances vary only minimally, which could also have an effect on the emission band. The Ba2–O distances vary between 277.9(2) and 320.71(2) pm, whereby the Ba3–O distance ranges from 279.1(2) to 322.4(2) pm and the Ba4–O distances range from 275.1(2) to 324.5(2) pm. Because the average Ba–O/N distances (300.5 pm) and the polyhedron size (56.46 Å^3^) for the Ba1 site is similar compared to the Ba2 split position with average Ba–O distances of 300.0–300.3 pm and a polyhedron size of 54.27 Å^3^, the difference in the emission bands may be dominated by the nephelauxetic effect.^29,30^ The 5*d* electron-level lowering for the activator ion Eu^2+^ is stronger when it is coordinated by N^3–^ anions than by O^2–^ anions due to the higher formal charge of N^3–^ compared with O^2–^. Consequently, it leads to a red shift of the emission band, for example, regarding the Ba1 site. In the case of the Ba1 site, the relatively large fwhm is caused by the fact that the coordination sphere varies depending on the occupation of the N1/O1 site (inhomogeneous line broadening). However, because the coordination sphere of the Ba2 split position is exclusively built up by O^2–^ anions, the nephelauxetic effect is smaller resulting in a blue shift of the emission band due to a less energetic lowering of the 5*d* levels. Based on the nephelauxetic effect and considering the different coordination sphere of the Ba1 site and the split position Ba2 site, it would indicate that the Ba1 site seems reasonable for the emission band at λ_max_ = 574 nm (2.18 eV) with a fwhm of 127 nm and the Ba2 site, including the Ba3 and Ba4 site, can attribute to the emission band at λ_max_ = 515 nm (2.41 eV) with a fwhm of 70 nm. Because especially the fwhm of Eu^2+^-based emission are temperature-dependent, the values of the luminescence properties of the title compound as well as the following values of the compounds known from literature are related to room temperature.

Although no barium oxonitridolithoaluminate is known so far, there are some representatives known in the literature with Sr^2+^ as central cation, such as Sr[Li_2_Al_2_O_2_N_2_]^1^ and Sr[Li_2.5_Al_1.5_O_3_N]^2^ for instance. In addition, the pure nitride and oxide variants such as Sr[LiAl_3_N_4_],^9^ Sr_*x*_Li_2+*x*_Al_2–*x*_O_4_,^31^ and Sr_2_LiAlO_4_^10^ could also be synthesized, and all these compounds could be successfully doped with the activator ion Eu^2+^. The variant Sr[Li_2_Al_2_O_2_N_2_]:Eu^2+^ crystallizes in the tetragonal space group *P*4_2_/*m* and has one Sr site, which is coordinated by four O^2–^ and four N^3–^ anions with Sr–O distances of 265.9(4) pm and Sr–N distances of 276.5(5) pm, resulting in a narrow band emission at 614 nm with a fwhm of 48 nm (1286 cm^–1^/0.1549 eV). The compound Sr[Li_2.5_Al_1.5_O_3_N]:Eu^2+^ crystallizes in the tetragonal space group *I*4/*m* and has one Sr site, which is coordinated by eight equidistant O/N sites with Sr–O/N distances of 267.4(4) pm, resulting in an emission maximum at 578 nm with a fwhm of 80 nm (2380 cm^–1^). In relation to the pure nitride variant Sr[LiAl_3_N_4_]:Eu^2+^, which crystallizes in the triclinic space group *P*1̅, there are two crystallographically distinguishable Sr sites in the structure and both are eightfold coordinated by N^3–^ anions. Because their polyhedron sizes only differ minimally with *V* = 34.26 Å^3^ for the Sr1 site and *V* = 34.00 Å^3^ for the Sr2 site, the compound exhibits only one narrow band emission at 650 nm with a fwhm of 50 nm (1180 cm^–1^/0.1463 eV). The compound Sr_*x*_Li_2+*x*_Al_2–*x*_O_4_:Eu^2+^ crystallizes, depending on the *x* value, in the tetragonal space group *P*4/*n* or in the superspace group *I*4/*m*(00γ)00. Because the Sr^2+^ cations are subject to a statistical disorder in the location of Sr^2+^ ions in the structure, a modulated structure model was applied. The Sr^2+^ cations are distributed at seven sites, some of which are partially occupied. All different Sr sites are coordinated by eight O^2–^ anions forming a slightly distorted cuboid with Sr–O distances varying between 254.4 and 296.4 pm regarding space group *P*4/*n*. The luminescence properties were investigated for the composition SrLi_3_AlO_4_:Eu^2+^ resulting in an emission maximum at 570 nm with a fwhm of ∼46 nm (∼1540 cm^–1^/∼0.19 eV).^32^ Due to the comparable structure of the four Sr variants, they show that a (partly) substitution of N by O and longer Sr–O/N distances leads to a blue shift in the emission profile.

The polymorphic compound Sr_2_LiAlO_4_:Eu^2+^ crystallizes not only in the monoclinic space group *P*2_1_/*m* but also in the orthorhombic space group *Cmcm*. The latter shows one emission maximum at 565 nm with a fwhm of 58 nm (2230 cm^–1^/0.28 eV) due to one Sr site in the structure with Sr–O distances of 266.8(5) pm. The former has two crystallographically distinguishable Sr sites with Sr2–O distances of 266.7(6) pm and Sr1–O distances of 269.4(5) pm resulting in a double band emission at 507 nm and 562 nm. The Sr1 site in Sr_2_LiAlO_4_:Eu^2+^ could be partly substituted by Ba^2+^ cations and has an effect on the luminescence properties. The compound Sr_1.85_Ba_0.15_LiAlO_4_:Eu^2+^ crystallizes in the orthorhombic space group *Pnma* with Sr1/Ba1–O distances of 272.1(9) pm and Sr2–O distances of 265.8(5) pm, which is in comparison to the Sr1–O distances and Sr2–O distances in the pure Sr variant larger and smaller, respectively. Because the compound shows no split emission band and exhibits only one emission maximum at 588 nm with a fwhm of 77 nm (2250 cm^–1^/0.28 eV), it can be assumed that only the Sr2 site is substituted by the activator ion Eu^2+^ due to the smaller Sr–O distances resulting in a red shift caused by a larger crystal field splitting. Due to the larger ionic radii of Ba^2+^ in contrast to Sr^2+^ cations {*r*[Ba^2+^ (CN = 8)] = 1.56 pm, *r*[Sr^2+^ (CN = 8)] = 1.40 pm},^33^ the *M*–O/N (*M* = Ba, Sr) distances of the Ba^2+^ cations are larger on average and thus a blue shift in the emission will be expected when Ba instead of Sr is incorporated into the structure.

In the literature, there are some known oxonitride phosphors that have Ba^2+^ as the central cation, such as Ba_3_Si_6_O_12_N_2_:Eu^2+^,^23,34^ Ba_3_Si_6_O_9_N_4_:Eu^2+^,^35,36^ BaSi_2_O_2_N_2_:Eu^2+^,^37,38^ BaAlSi_4_O_3_N_5_:Eu^2+^,^39^ BaAl_2_Si_3_O_4_N_4_:Eu^2+^,^40^ BaAlSi_5_N_7_O_2_:Eu^2+^,^41,42^ and BaSi_3_Al_3_O_4_N_5_:Eu^2+^.^43^ In the aforementioned compounds, the different Ba sites are possible positions to host the activator ion Eu^2+^ and their coordination spheres are presented in more detail below. The structure of Ba_3_Si_6_O_12_N_2_:Eu^2+^ is described by two crystallographically distinguishable Ba sites, which have different coordination spheres. The Ba1 site is coordinated by six O^2–^ anions with Ba–O distances of 277.4(3) pm, while the Ba2 site forms a coordination sphere to six O^2–^ anions and two N^3–^ anions with Ba–O distances between 281.9(3) and 290.2(3) pm and Ba–N distances ranging from 299.7(6) to 347.1(6) pm. Because this compound exhibits only one emission maximum at 527 nm (fwhm = 65 nm), only one of the two Ba sites is preferred for the incorporation of the activator ion Eu^2+^, which could be assigned to the Ba2 site. The effect of N^3–^ and O^2–^ anions on the emission maximum could be shown in the compound Ba_3_Si_6_O_9_N_4_:Eu^2+^ exhibiting one strong emission maximum at 520 nm (fwhm = 78 nm) and two weak emission maxima at λ_max_ = 370 nm, and 405 nm. According to Wei et al.,^35^ the strong emission maximum could be assigned to the Ba3 site building a coordination sphere to six O^2–^ anions and three N^3–^ anions, where the Ba–O distances range from 266.8(5) to 287.0(4) pm and the Ba–N distances are 341.2(1) pm. The Ba2 site, responsible for the emission maximum at λ_max_ = 450 nm, is coordinated by six O^2–^ anions, where the Ba–O distances ranging from 263.2(2) to 285.3(1) pm, and to two N^3–^ anions with Ba–N distances between 318.0(2) and 360.2(6) pm. The Ba1 site forms a coordination sphere to nine O^2–^ anions with Ba–O distances between 270.85(1) and 322.1(3) pm and is correlated to the emission maximum at λ_max_ = 370 nm. In this compound, a blue shift in the emission profile could be observed by the partial substitution of N^3–^ by O^2–^ anions. Furthermore, it could also be possible that the two minor emission bands in Ba_3_Si_6_O_9_N_4_:Eu^2+^ originate from, for example, defect luminescence, which could be clarified by decay time measurements.

The nephelauxetic effect on the emission bands can also be seen with the representatives BaAlSi_4_O_3_N_5_:Eu^2+^ and BaAl_2_Si_3_O_4_N_4_:Eu^2+^ in the field of barium oxonitridoalumosilicates. Because they have comparable structures, a red shift in the emission band can be observed with the increase in number of N^3–^ anions in the coordination sphere of the Ba^2+^ cations. Both compounds crystallizes in the orthorhombic space group *A*2_1_*am* having one Ba site. The Ba site in the compound BaAl_2_Si_3_O_4_N_4_:Eu^2+^ builds up a coordination sphere to three N^3–^ and five O^2–^ anions with Ba–O distances between 275.9 and 322.0 pm and Ba–N distances ranging from 306.7 to 333.0 pm. The compound exhibits an observed emission maximum at 458 nm (fwhm = ∼100 nm). The Ba site in the variant BaAlSi_4_O_3_N_5_:Eu^2+^ is coordinated by four O^2–^ and five N^3–^ anions with Ba–O distances varying between 285.6(7) and 286.1(7) pm and Ba–N distances ranging from 312.5(8) and 321.1(4) pm. This oxonitride variant exhibits an emission maximum at 475 nm (fwhm = ∼100 nm).

Different structures to the aforementioned barium oxonitridoalumosilicates show the compounds BaAlSi_5_N_7_O_2_:Eu^2+^ and BaSi_3_Al_3_O_4_N_5_:Eu^2+^. The representative with the composition BaSi_3_Al_3_O_4_N_5_:Eu^2+^ crystallizes in the monoclinic space group *P*2_1_/*m* with one Ba site, which is coordinated by four O^2–^ and four N^3–^ anions with Ba–O distances in a range of 261.8(7) and 272.0(4) pm and Ba–N distances between 274.8(7) and 331.2(8) pm resulting in an emission maximum at 470 nm (fwhm = ∼75 nm). In the structure of BaAlSi_5_N_7_O_2_:Eu^2+^, which crystallizes in the orthorhombic space group *Imm*2, there is one Ba site coordinated by four O^2–^ and six N^3–^ anions with Ba–O distances between 285.1(7) and 335.0(7) pm and Ba–N distances ranging from 319.7(7) to 336.7(5) pm, resulting in an observed broad band emission maximum at 515 nm (fwhm = ∼100 nm).

Furthermore, the compound BaSi_2_O_2_N_2_:Eu^2+^ features a layered structure, which can crystallize in two different orthorhombic space groups *Cmcm* and *Pbcn*. In both structure models is one Ba site, which is cuboid-like coordinated by eight O^2–^ anions. The resulting cuboid is additionally capped by two N^3–^ anions with Ba–O distances between 273(1) and 308.8(9) pm and Ba–N distances ranging from 329.8(6) to 334.3(6) pm resulting in an observed emission maximum at 490–500 nm (fwhm = ∼40 nm). In comparison to the phosphors with Ba^2+^ as the central cation, the Ba–O/N distances in the compound Ba_4_Al_7_Li_28.08_O_26.92_N_1.08_ are in a similar range. Furthermore, the nephelauxetic effect can be observed for comparable structures or in structures with more than one Ba site, which forms different coordination spheres to O^2–^ and N^3–^ anions, implying the assignment of the emission bands to the different Ba sites seems consistent in the here presented compound. An overview of all aforementioned compounds are represented in Table S3 in the Supporting Information.

## Conclusions

This work contains a detailed discussion of the crystal structure and luminescence properties of the first barium oxonitridolithoaluminate Ba_4_Al_7_Li_28.08_O_26.92_N_1.08_. The anionic part of the structure is built up by AlO_3_N/AlO_4_ tetrahedra and highly condensed edge- and corner-sharing LiO_4_ tetrahedra forming seven-membered rings and a cross-linked framework. The 10-fold coordinated Ba^2+^ cations are incorporated between the seven-membered rings and form polyhedra chains along the crystallographic *b*-axis. The connection pattern in the anionic framework of LiO_4_ and AlO_4_ tetrahedra could not be observed in any known compound yet resulting in a new structure type. Due to the high proportion of Li^+^ cations in the title compound, this results in a degree of condensation of ∼1.25 and represents the first barium oxonitridolithoaluminate with such a characteristic. In addition, Ba_4_Al_7_Li_28.08_O_26.92_N_1.08_ could successfully be doped with the activator ion Eu^2+^ and shows a broad band emission in the green to yellow spectral region at λ_max_ = 524 nm (2.34 eV) with a fwhm of 112 nm (4373 cm^–1^/0.54 eV), which can be described by a superposition of two adjusted emission bands at λ_max_ = 515 nm (2.41 eV) with a fwhm of 70 nm (2704 cm^–1^/0.34 eV), and at λ_max_ = 574 nm (2.18 eV) with a fwhm of 127 nm (4127 cm^−1^/0.51 eV). By comparing the Ba–O/N distances and the different coordination spheres of the two crystallographically distinguishable Ba sites with already known compounds from the literature, it was possible to assign the observed emission bands to the respective Ba sites. In comparison to the known luminescent barium oxonitridosilicates and garnets such as Lu_3_Al_5_O_12_:Ce^3+^,^44^ the emission band is in a similar spectral region, but the title compound can be synthesized at much lower temperatures. Due to the lower stability and longer synthesis time in relation to the aforementioned compounds, further optimization of the compound Ba_4_Al_7_Li_28.08_O_26.92_N_1.08_:Eu^2+^ regarding synthesis and stability have to be done for any industrial application. Because the LiO_4_ tetrahedra form a similar highly condensed framework as comparatively the beryllates; for instance, this could lead to the discovery of new phosphors in the field of (oxonitrido)lithoaluminates and also -silicates, which could result in interesting luminescence properties.

## Experimental Section and Theoretical Methods

### Synthesis

Single-crystals of the compound Ba_4_Al_7_Li_28.08_O_26.92_N_1.08_ could be synthesized by a mixture of BaO (56.60 mg, 0.369 mmol, Sigma-Aldrich >99.99%), Li_2_CO_3_ (73.89 mg, 0.185 mmol, Fluka >99.5%), Al_2_O_3_ (18.82 mg, 0.185 mmol, Alfa Aesar >99.9%), Li_3_N (9.64 mg, 0.277 mmol, Alfa Aesar >99.4%), Eu_2_O_3_ (1.30 mg, 0.004 mmol, Smart Elements >99.99%), and lithium metal (4.82 mg, 0.694 mmol, Sigma-Aldrich >99%) in a molar ratio of 4:2:2:3:0.04:7.5. The starting materials were ground in an agate mortar until a homogenous mixture was obtained, filled in a tantalum ampoule (30 mm length, 9.5 mm diameter, 0.5 mm wall thickness) and sealed via arc-welding under a pressure of 400 mbar. During the arc-welding, the crucible holder was cooled with water to avoid chemical reactions. All preparation steps were carried out using an inert gas (Ar 5.0, Messner Austria GmbH)-filled glovebox (MBraun, O_2_ < 1 ppm, H_2_O < 1 ppm) due to the moisture sensitivity of the starting material (Li and Li_3_N). The tantalum ampoule was placed into a silica-glass tube, which was filled with 400 mbar argon, heated to 830 °C within 7 h, maintained at that temperature for 200 h, and cooled down to room temperature by a cooling rate of 0.1 °C/min. The yielded inhomogeneous white powder sample contained a few crystals, from which one of them was identified as the title compound (see Figure S1 in the Supporting Information). Experiments to obtain the compound as the main phase have not been successful so far and the single-crystals decompose within a few days.

### Single-Crystal X-ray Diffraction

The reaction product was analyzed with a polarization contrast microscope to identify suitable single-crystals, which were isolated for X-ray measurements on a Bruker D8 Quest diffractometer (Mo-*K*α radiation, λ = 0.7107 Å) equipped with a Photon 100 CMOS detector. Data processing and multi-scan absorption correction were carried out with the programs SADABAS^45^ and SAINT.^46^ For structure solution and refinement, the SHELXS/L^47,48^ software implemented in the program WINGX^49^ was applied. Further details of the crystal structure investigation on a single-crystal of Ba_4_Al_7_Li_28.08_O_26.92_N_1.08_ may be obtained from The Cambridge Crystallographic Data Centre via www.ccdc.cam.ac.uk/structures/ under the deposition number 2205834.

### Luminescence

A setup of a blue laser diode (λ = 448 nm, THORLABS, Newton, NJ, USA) in combination with a CCD Detector (AVA AvaSpec 2048, AVANTES, Apeldoorn, Netherlands) was used to record the emission spectra of the green-yellow luminescent Ba_4_Al_7_Li_28.08_O_26.92_N_1.08_ single-crystal. The data preparation was carried out by the software AVA AVASOFT (version 7).

## Supporting Information Available

Anisotropic displacement parameters of the crystal structure, interatomic distances in Ba_4_Al_7_Li_28.08_O_26.92_N_1.08_, overview of reported phosphors in the luminescence section, and obtained agglomerates of Ba_4_Al_7_Li_28.08_O_26.92_N_1.08_:Eu^2+^ (PDF)
