# Supplementary material for: Ba4Al7Li28.08O26.92N1.08, the Barium Oxonitridolithoaluminate with a Highly Condensed LiO4 Tetrahedra Framework
Source: Inorg Chem. 2022 Dec 16;62(1):213–23. doi: 10.1021/acs.inorgchem.2c03211 (PMC9832532; doi:10.1021/acs.inorgchem.2c03211)
Supplement: Supplementary file 1 — ic2c03211_si_001.pdf [file ic2c03211_si_001.pdf]

Supporting Information

$Ba_4Al_7Li_{28.08}O_{26.92}N_{1.08}$, the barium oxonitridolithoaluminate with a highly condensed LiO_4 tetrahedra framework

Daniel S. Wimmer[a], Markus Seibald[b], Dominik Baumann[b], Klaus Wurst[a], and Hubert Huppertz*[a]

[a] Daniel S. Wimmer, Klaus Wurst, Hubert Huppertz:
Institut für Allgemeine, Anorganische und Theoretische Chemie,
Universität Innsbruck
Innrain 80-82, A-6020 Innsbruck, Austria
E-Mail: *Hubert.Huppertz@uibk.ac.at*
https://www.uibk.ac.at/aatc/mitarbeiter/hub/

[b] Markus Seibald, Dominik Baumann:
ams-OSRAM International GmbH,
Mittelstetter Weg 2, D-86830 Schwabmünchen, Germany

Table S1: Anisotropic displacement parameters U_{ij} (Å²) of $Ba_4Al_7Li_{28.08}O_{26.92}N_{1.08}$ (standard deviations in parentheses). The displacement parameters of the atoms Ba2, Ba3, and Ba4 were refined isotropically.

Atom	U_{11}	U_{22}	U_{33}	U_{23}	U_{13}	U_{12}
Ba1	0.01556(7)	0.01114(6)	0.01062(6)	0	0.00419(4)	0
Al1	0.0058(2)	0.0070(2)	0.0044(2)	0	-0.0001(2)	0
Al2	0.0050(2)	0.0070(2)	0.0044(2)	0	0.0003(2)	0
Al3	0.0053(2)	0.0058(2)	0.0052(2)	0	0.0005(2)	0
Al4	0.0047(4)	0.0071(4)	0.0051(4)	0	0.0002(3)	0
Al5	0.015(2)	0.011(2)	0.007(2)	-0.0030(9)	0.0004(8)	-0.0016(9)
Li1	0.007(2)	0.016(2)	0.009(2)	-0.003(2)	0.000(2)	-0.002(2)
Li2	0.015(2)	0.011(2)	0.007(2)	-0.0010(9)	0.0018(9)	-0.0008(9)
Li3	0.0053(2)	0.0058(2)	0.0052(2)	0	0.0005(2)	0
Li4	0.0047(4)	0.0071(4)	0.0051(4)	0	0.0002(3)	0
Li5	0.011(2)	0.012(2)	0.008(2)	0	0.001(2)	0
Li6	0.014(2)	0.015(2)	0.011(2)	0	0.002(2)	0
Li7	0.011(2)	0.014(2)	0.012(2)	0.0012(9)	0.0005(9)	0.0006(9)
Li8	0.011(2)	0.010(2)	0.008(2)	-0.0002(8)	0.0001(8)	0.0013(9)
Li9	0.022(2)	0.024(2)	0.018(2)	-0.002(2)	-0.003(2)	0.002(2)
Li10	0.010(2)	0.010(2)	0.015(2)	0	0.003(2)	0
Li11	0.018(2)	0.009(2)	0.011(2)	0	0.004(2)	0
O1	0.0075(7)	0.0224(9)	0.0116(7)	0	-0.0034(5)	0
O2	0.0137(7)	0.0149(7)	0.0079(6)	0	0.0042(5)	0
O3	0.0075(6)	0.0186(7)	0.0124(6)	-0.0016(6)	0.0019(5)	-0.0026(5)
O4	0.0081(6)	0.0079(6)	0.0097(6)	0	-0.0001(5)	0
O5	0.0079(6)	0.0089(6)	0.0097(6)	0	-0.0016(5)	0
O6	0.0109(6)	0.0082(6)	0.0069(6)	0	-0.0002(5)	0
O7	0.0097(4)	0.0076(4)	0.0073(4)	-0.0005(3)	0.0008(3)	-0.0008(3)
O8	0.0103(4)	0.0088(4)	0.0105(4)	-0.0016(3)	0.0014(3)	-0.0017(3)
O9	0.0126(6)	0.0076(6)	0.0056(6)	0	0.0010(5)	0
O10	0.0160(7)	0.0235(8)	0.0067(6)	0	0.0054(5)	0
O11	0.0186(5)	0.0130(5)	0.0124(5)	0.0034(4)	0.0058(4)	0.0079(4)
N1	0.0075(7)	0.0224(9)	0.0116(7)	0	-0.0034(5)	0

Table S2: Interatomic distances (Å) in $Ba_4Al_7Li_{28.08}O_{26.92}N_{1.08}$ (standard deviations in parentheses).

Ba1–O3	2.8607(2) 2×	Al1–O11	1.769(2) 2×	Li2–O11	1.914(3)
Ba1–O2	2.917(2)	Al1–O2	1.819(3)	Li2–O4	1.945(3)
Ba1–O4	2.951(2)	**Ø Al1–O**	**1.786(3)**	Li2–O5	1.960(3)
Ba1–O8	3.019(2) 2×			Li2–O7	2.148(3)
Ba1–O11	3.109(2) 2×	Al1–N1/O1	1.798(2)	**Ø Li2–O**	**1.992(3)**
**Ø Ba1–O**	**2.981(2)**	**Ø Al1–N1/O1**	**1.798(2)**		
				Li6–O6	1.955(4)
Ba1–N1/O1	3.1007(4) 2×	Al2–O10	1.754(2)	Li6–O10	1.993(4)
**Ø Ba1–N/O**	**3.1007(4)**	Al2–O8	1.777(2) 2×	Li6–O7	2.079(3) 2×
		**Ø Al2–O**	**1.769(2)**	**Ø Li6–O**	**2.027(4)**
Ba2–O6	2.779(2)				
Ba2–O7	2.811(2) 2×	Al2–N1/O1	1.822(3)	Li7–O8	1.900(3)
Ba2–O2	2.858(2)	**Ø Al2–N1/O1**	**1.822(3)**	Li7–O9	1.978(3)
Ba2–O8	3.020(2) 2×			Li7–O9	2.028(3)
Ba2–O11	3.141(2) 2×	Al3/Li3–O4	1.762(2)	Li7–O7	2.040(3)
Ba2–O10	3.2071(5) 2×	Al3/Li3–O7	1.793(2) 2×	**Ø Li7–O**	**1.987(3)**
**Ø Ba2–O**	**3.000(2)**	Al3/Li3–O9	1.805(2)		
		**Ø Al3/Li3–O**	**1.788(2)**	Li8–O6	1.959(3) 2×
Ba3–O2	2.791(2)			Li8–O7	2.046(3)
Ba3–O7	2.850(2) 2×	Al4/Li4–O5	1.856(2)	Li8–O11	2.131(3)
Ba3–O8	2.873(2) 2×	Al4/Li4–O3	1.8674(5) 2×	**Ø Li8–O**	**2.024(3)**
Ba3–O6	2.962(2)	**Ø Al4/Li4–O**	**1.864(2)**		
Ba3–O10	3.1803(5) 2×			Li9–O10	1.976(4)
Ba3–O11	3.224(2) 2×	Al4/Li4–N1/O1	1.926(2)	Li9–O2	2.024(4)
**Ø Ba3–O**	**3.001(2)**	**Ø Al4/Li4–N1/O1**	**1.926(2)**	Li9–O2	2.037(4)
				Li9–O11	2.144(4)
Ba4–O7	2.751(2)	Al5/Li5–O10	1.876(4)	**Ø Li9–O**	**2.045(4)**
Ba4–O2	2.782(2)	Al5/Li5–O11	1.924(2) 2×		
Ba4–O8	2.860(2)	Al5/Li5–O6	2.019(4)	Li10–O5	1.878(4)
Ba4–O8	2.862(2)	**Ø Al5/Li5–O**	**1.936(4)**	Li10–O7	1.962(3) 2×
Ba4–O10	2.927(2)			Li10–O9	2.014(4)
Ba4–O11	2.999(2)	Li1–O3	1.904(3)	**Ø Li10–O**	**1.954(4)**
Ba4–O7	3.028(2)	Li1–O4	1.929(3)		
Ba4–O8	3.1408(2)	Li1–O8	1.955(3)	Li11–O8	1.867(3) 2×
Ba4–O11	3.245(2)	Li1–O5	2.045(3)	Li11–O5	2.000(5)

Ba4–O10	3.439(2)	**Ø Li1–O**	**1.958(3)**	Li11–O9	2.177(5)
**Ø Ba4–O**	**3.003(2)**			**Ø Li11–O**	**1.978(5)**

Table S3: Overview of all compounds reported in the luminescence section with respect to the different Ba/Sr sites, coordination sphere, bond lengths, emission maxima, and fwhm.

Compound	Space group	Ba/Sr sites	Number of bonds to O	Number of bonds to N	M−O / pm	M−N / pm	Emission maximum / nm	fwhm / nm
$Sr[Li_2Al_2O_2N_2]:Eu^{2+}$	$P4_2/m$	Sr1	4	4	265.9(4)	276.5(5)	614	48
$Sr[Li_{2.5}Al_{1.5}O_3N]:Eu^{2+}$	$I4/m$	Sr1	8 equidistant O/N		267.4(4)		578	80
$Sr[LiAl_3N_4]:Eu^{2+}$	$P\overline{1}$	Sr1		8		279.7	650	50
		Sr2		8		280.3		
$Sr_xLi_{2+x}Al_{2-x}O_4:Eu^{2+}$	$P4/n$	Seven Sr sites	8		254.4–296.4		570	46
$Sr_2LiAlO_4:Eu^{2+}$	$Cmcm$	Sr1	8		266.8(5)		565	58
$Sr_2LiAlO_4:Eu^{2+}$	$P2_1/m$	Sr1	8		269.4(5)		507	
		Sr2	8		266.7(6)		562	
$Sr_{1.85}Ba_{0.15}LiAlO_4:Eu^{2+}$	$Pnma$	Sr1/Ba1	8		272.1(9)			
		Sr2	8		265.8(5)		588	77
$Ba_3Si_6O_{12}N_2:Eu^{2+}$	$P\overline{3}$	Ba1	6		277.4(3)			
		Ba2	6	2	281.9(3)–290.2(3)	299.7(6)–347.1(6)	527	65
$Ba_3Si_6O_9N_4:Eu^{2+}$	$P3$	Ba1	9		270.85(1)–322.1(3)		370	
		Ba2	6	2	263.2(2)–285.3(1)	318.0(2)–360.2(6)	450	
		Ba3	6	3	266.8(5)–287.0(4)	341.2(1)	520	78
$BaAlSi_4O_3N_5:Eu^{2+}$	$A2_1am$	Ba1	4	5	285.6(7)–286.1(7)	312.5(8)–321.1(4)	475	~100
$BaAl_2Si_3O_4N_4:Eu^{2+}$	$A2_1am$	Ba1	5	3	275.9–322.0	306.7–333.0	458	~100
$BaAlSi_5N_7O_2:Eu^{2+}$	$Imm2$	Ba1	4	6	285.1(7)–335.0(7)	319.7(7)–336.7(5)	515	~100

$BaSi_3Al_3O_4N_5:Eu^{2+}$	*P*2$_1$/*m*	Ba1	4	4	261.8(7)–272.0(4)	274.8(7)–331.2(8)	470	~75
$BaSi_2O_2N_2:Eu^{2+}$	*Cmcm* / *Pbcn*	Ba1	8	2	273(1)–308.8(9)	329.8(6)–334.3(6)	490–500	~40

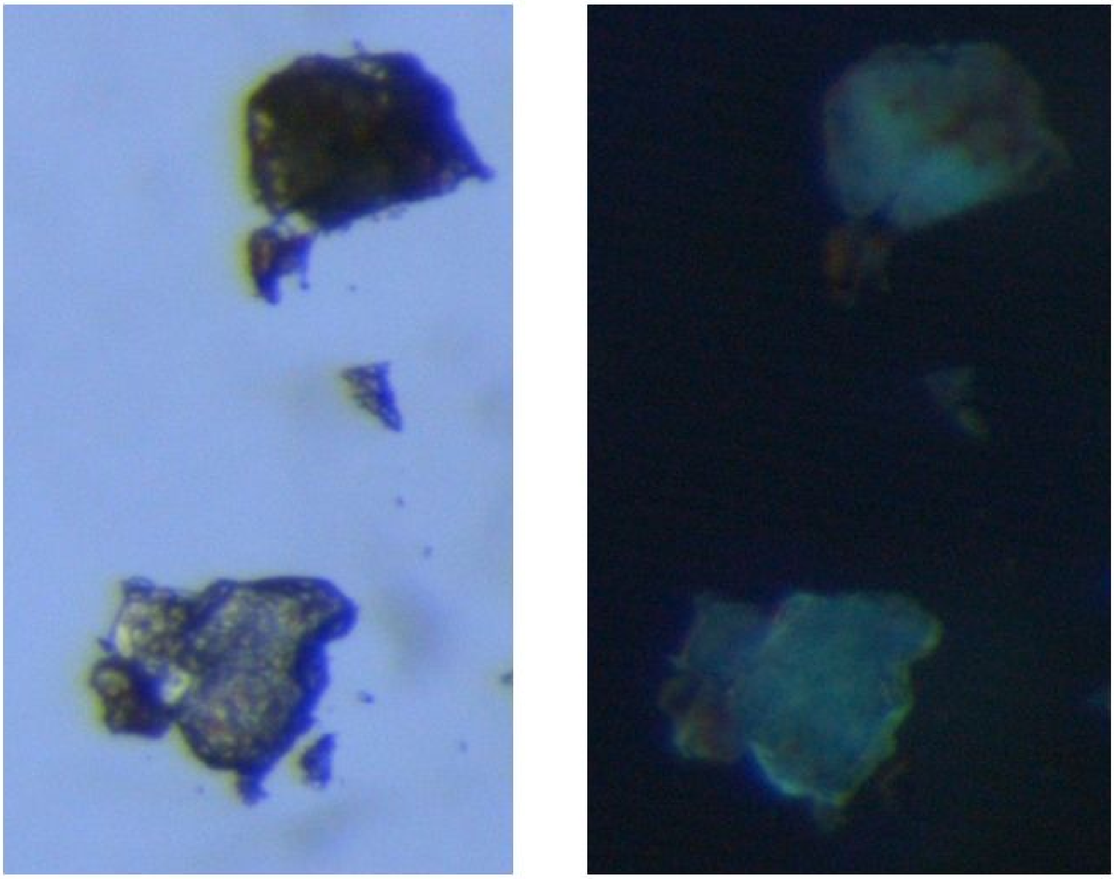

Figure S1: The obtained agglomerates of the compound $Ba_4Al_7Li_{28.08}O_{26.92}N_{1.08}:Eu^{2+}$. Left: Optical appearance of the compound under daylight. Right: Optical appearance of the compound under UV-light.
